# A 33-Year-Old Man with Progressive Diffuse and Focal Neuropsychiatric Signs without Localizing Correlates on Brain Imaging: A Systematic Approach to Diagnosis

**DOI:** 10.26502/jppd.2572-519x0113

**Published:** 2020-09-09

**Authors:** Khaled Moussawi, Taha Gholipour, Vani Rao

**Affiliations:** 1 National Institute on Drug Abuse, Baltimore, MD, USA; 2 Department of Neurology, The George Washington University, Washington, DC; 3 Johns Hopkins Medicine, Baltimore, MD, USA

**Keywords:** Dementia, Neurosyphilis, General paresis

## Abstract

**Introduction:**

Novel expensive diagnostic tests are rapidly emerging. However, the answer to the most complex clinical presentations is often inferred from a systematic approach to the differential diagnosis. This is especially the case in neuropsychiatric disorders that present with a mix of neurologic and psychiatric symptoms. This case report fills a gap in the literature by providing a systematic differential diagnosis of such neuropsychiatric presentations associated with non-focal brain imaging.

**Case Presentation:**

A 33-year-old African-American man presented with confusion, weakness, and tremors. He initially noted memory problems and over the following six months progressively became confused, developed speech difficulties and left sided weakness and tremors. On exam, he was predominantly abulic but with intermittent and extreme mood lability. He lacked insight and his attention was poor. He had mild facial weakness and spastic hemiparesis with action tremors on the left side. Magnetic Resonance Imaging of the brain demonstrated non-specific diffuse parenchymal volume loss. His serum and cerebrospinal fluid studies were positive for Rapid Plasma Reagin and Veneral Disease Research Laboratory tests, respectively, suggesting a diagnosis of paretic neurosyphilis.

**Conclusion:**

This is a case of a young man with neurosyphilis who presented with progressive subacute cognitive decline, associated with focal neurological signs but no focal lesions on brain imaging. Neurosyphilis is often misdiagnosed on medicine, psychiatry, and neurology inpatient units. In this report, we present an approach to conceptualize similar cases and provide a differential diagnosis that will help reach an accurate diagnosis more efficiently. Further, it raises awareness regarding neurosyphilis, a devastating but easily treatable condition.

## 1. Introduction

With new knowledge and new technologies rapidly emerging, the repertoire of diagnostic tests and associated medical costs are ballooning. Further, every diagnostic test inherently carries a risk of false positives and incidental findings, which result in misdiagnoses and unnecessary further workups. This has become a challenge in fields like neurology and psychiatry, where genetic sequencing, paraneoplastic panels, and sophisticated imaging modalities are becoming routine diagnostic tests. However, the answer to the most complex clinical presentations is often inferred from a systematic approach to the differential diagnosis. This is especially the case in neuropsychiatric disorders that present with a mix of neurologic and psychiatric symptoms. This case report fills a gap in the literature by providing a systematic differential diagnosis of such neuropsychiatric presentations associated with non-focal brain imaging.

## 2. Case Presentation

A 33-year-old African-American male presented to the hospital with confusion, weakness, and tremors. His symptoms started six months earlier with memory impairment followed by auditory hallucinations, bizarre behavior, and grandiose delusions, for which he received neuroleptics without improvement. He gradually developed speech difficulties, unsteady gait, and left-sided tremors and weakness. He was hospitalized multiple times without a clear diagnosis, and by the time of presentation, he was withdrawn and not engaging in meaningful activities or conversations.

He was born and raised in a rural area. His birth and developmental history were unremarkable and he had no behavioral or learning problems. He lived with his wife of nine years and their two daughters who were all in good health. He had no significant past medical or psychiatric history besides his present illness and was not on any medications at the time of presentation. He smoked cigarettes daily and marijuana occasionally but denied any history of other drug use.

On exam, he was alert and calm. He appeared mostly abulic with flat affect, psychomotor slowing, and no spontaneous speech. His answers were one to two words, appropriate but unreliable. Occasionally, his abulia remitted and he became very emotional and labile. He would start crying and express feelings of hopelessness and fear of dying, but then over the course of few minutes, he would become jovial, disinhibited, his behavior puerile, and his mood expansive. He scored 13/30 on the Montreal Cognitive Assessment test (MoCA). His deficits involved multiple domains including executive, visuospatial, and memory.

On neurologic exam, he had bilateral ptosis and a mydriatic right pupil that was poorly reactive to light and accommodation. The extraocular movements were intact. He had a mild left lower facial weakness, subtle left spastic hemiparesis, and hemibody action and postural tremor of high amplitude and moderate frequency. His reflexes were diffusely brisk, though more prominently on the left. He had a present left Babinski sign. Pinprick sensation was reduced in the left leg. His gait was spastic-hemiparetic.

**Figure 1:**
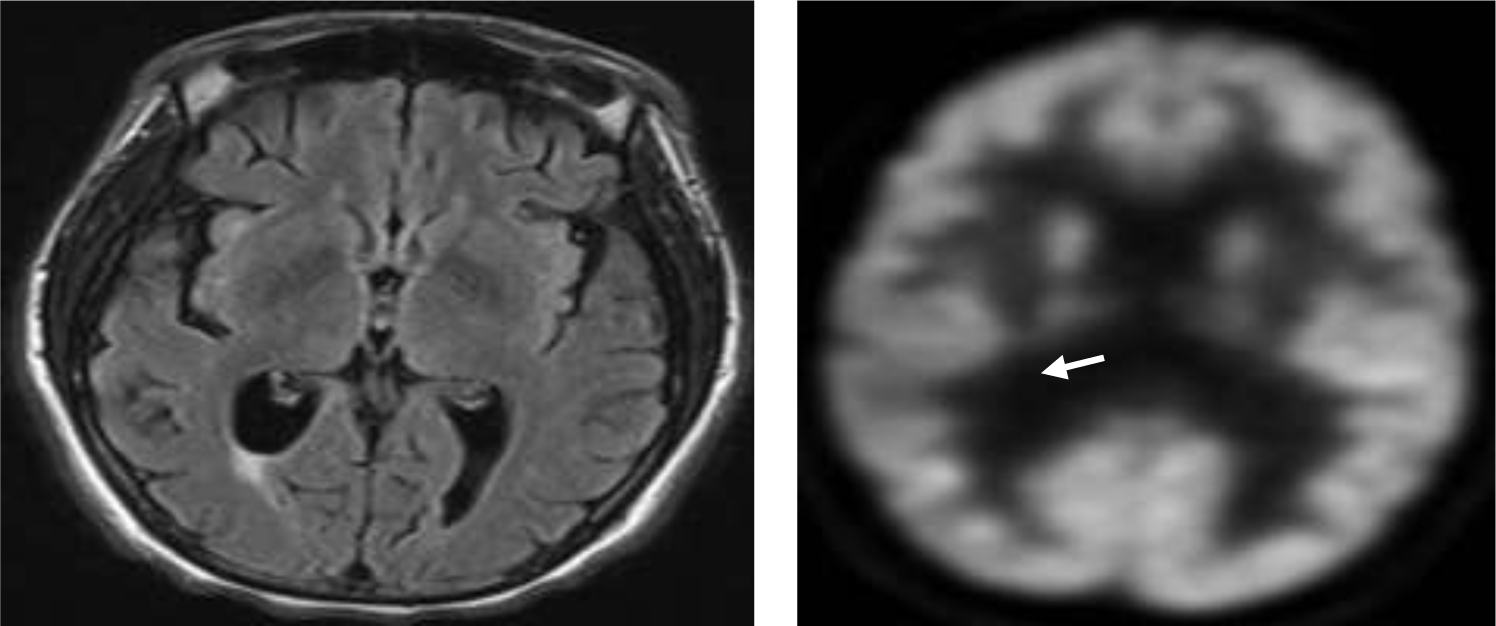
**(A)** Axial brain MRI FLAIR sequence illustrating diffuse atrophy and non-specific white matter changes: (**B)** Brain FDG-PET scan showing cortical hypometabolism in the right parietal lobe (white arrow).

Initial workup, including complete blood count and comprehensive metabolic panel, was normal. Toxicology screen was positive for oxycodone and cannabinoids. Brain magnetic resonance imaging (MRI) demonstrated mild diffuse parenchymal volume loss and subtle nonspecific white matter changes (Figure1, A), without abnormal focal lesions or enhancement. A brain magnetic resonance angiogram (MRA) was normal.

## 3. Discussion

Briefly, this patient presented with rapidly progressive behavioral, emotional, and cognitive decline, associated with focal neurological signs and non-focal brain imaging. It is tempting to localize the left hemiparesis and action tremor as well as the right mydriasis to a single ventral midbrain lesion ipsilateral to the mydriatic pupil. However, the intact extraocular movements, severe encephalopathy, and non-focal brain MRI all suggest a more diffuse process. Multiple dimensions can be invoked to aid with the differential diagnosis in this case, namely 1) age-of onset, 2) time course of progression, 3) symptoms associated with the behavioral and cognitive decline, and 4) the presence of focal neurological signs in the absence of anatomically correlated findings on brain imaging.

The first dimension is the age of onset, which helps categorize the case as a *young-onset dementia* (i.e., patients younger than 65 years) [1]. In this age group, Alzheimer disease, vascular dementia, and frontotemporal dementia remain the most common (34%, 18%, and 12% respectively) [2]. In addition, the differential diagnosis for young onset dementias is extensive and includes other primary neurodegenerative diseases, inflammatory/autoimmune, neoplastic, infectious, metabolic, epileptic, genetic, and toxic causes. Of note, closer dementia onset to adolescence or early adulthood makes it more likely to be from genetic or metabolic causes [1].

The second dimension to invoke is the rate of progression, which places this case in the *rapidly progressive dementias* category. These are defined as “conditions that progress from onset of first symptom to dementia in less than one to two years” [3]. The differential diagnosis for this group is also extensive and includes neurodegenerative (including Alzheimer’s disease), vascular, inflammatory/autoimmune, neoplastic, infectious, metabolic, epileptic, genetic, and toxic causes [3]. In fact, survey of the literature reveals that the differential diagnosis for young onset and rapidly progressive dementias is very similar, almost identical [3,4].

Another dimension is to conceptualize the case as a *“dementia-plus syndrome”.* This term was coined by Sampson et. al to refer to young-onset dementias where cognitive decline is associated with neurological or systemic disturbances [1]. This construct is very useful in narrowing and guiding the diagnostic investigation. Rossor et. al provide a detailed and very helpful differential diagnosis for the dementia-plus syndromes organized by neurological or systemic features [2].

Finally, one can approach the case along the dimension of *encephalopathies with focal neurological signs but without anatomical correlates on brain imaging*. With the advances in brain imaging especially brain MRI, few encephalopathies present with focal neurological signs and non-focal MRI findings. Hence, we generated a differential diagnosis of similar presentations (Table1).

Thinking along these dimensions, and examining the differential listed in Table 1, the specific presentation of our patient (progressive encephalopathy, multiple neuropsychiatric symptoms, and focal neurological signs) raises the suspicion for certain infectious diagnoses like paretic neurosyphilis or “general paresis of the insane” [5].

Further workup, including HIV serology, ammonia, TSH, vitamin B12, and folate levels, was unremarkable. Routine EEG was normal without slowing, but brain FDG-PET scan showed cortical hypometabolism in the right parietal lobe, right inferior frontal gyrus, right insula, and bilateral temporal lobes (Figure 1, B). Treponemal antibody testing was positive, and the rapid plasma reagin (RPR) was positive at a titer of 1:128, which was diagnostic of systemic syphilis and suggestive of neurosyphilis [6]. To confirm the latter, a lumbar puncture was performed, which showed normal opening pressure, lymphocytic pleocytosis (40 WBC/mm^3^, 90% lymphocytes), elevated protein (75mg/dL), and positive VDRL at a titer of 1:32. These results, along with the clinical presentation, are diagnostic of paretic neurosyphilis [6,7].

The rate of syphilis infection has been steadily increasing over the past few years, resulting in higher neurosyphilis presentations, especially in patients infected with HIV [6]. Neurosyphilis is referred to as the great imitator due to its varied presentations (neuropathy, myelopathy, meningitis, ischemic strokes, seizures, psychosis, depression, mania, anxiety, personality changes, cognitive decline, and ocular problems of any kind) [5,8,9]. It is caused by the *Treponema pallidum* spirochetes’ invasion of the central nervous system (neuroinvasion) which occurs early in infection [10,11]. It can be asymptomatic and clear spontaneously, or result in one or more of many clinical syndromes: 1) meningeal neurosyphilis presenting as meningitis within one year after infection; 2) vascular neurosyphilis from vasculitis of small, medium, and large vessels, presenting with ischemic strokes in the first 10 years after infection; 3) paretic neurosyphilis from brain parenchymal invasion presenting after 10–20 years; and 4) tabes dorsalis from dorsal column and spinal nerve root invasion presenting after 25–30 years [8,10].

Kraeplin in his monologue described in great detail the different neuropsychiatric manifestations of paretic neurosyphilis (general paresis) [5]. In addition to focal neurological signs and symptoms, paretic neurosyphilis most often presents with cognitive decline and myriad psychiatric symptoms including depression, mania, anxiety, delusions, hallucinations, change in behavior or personality, apathy, and irritability [5,12]. Unfortunately, recent studies indicate that paretic neurosyphilis is often missed initially in admitted patients (83–100% misdiagnosis rate) [13,14]. Initial misdiagnoses include dementia, delirium, schizophrenia, Parkinson disease, viral encephalitis, depression, mania, epilepsy, or alcohol related complications [13,14]. Brain imaging in paretic neurosyphilis is non-suggestive and usually shows diffuse cerebral atrophy and non-specific T2-hyperintensities on brain MRI [15].

Our patient was treated with intravenous penicillin per the CDC guidelines [6]. His wife tested negative for syphilis. The patient had to move out of state back to his hometown. He was lost to follow-up and did not show up for a repeat lumbar puncture 6 months after treatment to assess for therapeutic response [6]. In general, treatment of paretic neurosyphilis halts the disease progression and results in some occupational adjustment in 35–40% of patients, while 40–50% remain dependent [8].

## 4. Conclusion

This is a case of a young man with paretic neurosyphilis who presented with progressive encephalopathy and neuropsychiatric symptoms associated with focal neurological deficits but no correlated lesions on brain MRI. Neurosyphilis is often misdiagnosed on inpatient units, delaying treatment of this devastating but treatable disease, which is becoming increasingly prevalent. In this report, we discussed a multi-dimensional approach and provided a differential diagnosis that fills a gap in the literature regarding similar neuropsychiatric presentations.

## Figures and Tables

**Table 1:** Partial differential diagnosis for encephalopathies with focal neurological signs but without anatomical correlates on brain MRI

**Neurodegenerative**	Corticobasal degeneration Progressive supranuclear palsy Frontotemporal dementia with motor neuron disease Prion disease Spinocerebellar ataxias
**Metabolic disorders**	Mithocondrial diseases (MELAS) Hyperammonemia Uremia Hypo/hyperglycemia Nutritional deficiencies
**Epilepsy Infections**	
**Bacetria**	Neurosyphilis Tuberculosis Whipple
**Fungi**	Cryptococcus
**Viruses**	Measles (SSPE) HIV Rubella
**Inflammatory/autoimmune**	Hashimoto encephalopathy Thrombotic therombocytopenic purpura (TTP) SLE Neurosarcoid Behcet Paraneoplastic syndromes
**Genetic**	Wilson’s Neuroacanthocytosis Late onset inborn erros of metabolism (Gaucher, Nieman-PickC) Fragile X-associated tremor ataxia syndrome

## Data Availability

Please contact corresponding author.
